# Diabetes Mellitus and Amyotrophic Lateral Sclerosis: A Systematic Review

**DOI:** 10.3390/biom11060867

**Published:** 2021-06-10

**Authors:** Laura Ferri, Paola Ajdinaj, Marianna Gabriella Rispoli, Claudia Carrarini, Filomena Barbone, Damiano D’Ardes, Margherita Capasso, Antonio Di Muzio, Francesco Cipollone, Marco Onofrj, Laura Bonanni

**Affiliations:** 1 Department of Neuroscience, Imaging and Clinical Sciences, University G. d’Annunzio of Chieti-Pescara, 66100 Chieti, Italy; ferrilaura@outlook.it (L.F.); paola.ajdinaj@gmail.com (P.A.); mariannarispoli92@gmail.com (M.G.R.); claudia.carrarini@live.it (C.C.); agapefilo@gmail.com (F.B.); onofrj@unich.it (M.O.); 2 Department of Medicine and Aging Sciences, University G. d’Annunzio of Chieti-Pescara, 66100 Chieti, Italy; damiano.med@libero.it (D.D.); francesco.cipollone@unich.it (F.C.); 3 Department of Neurology, “SS Annunziata” Hospital, 66100 Chieti, Italy; neurolab@unich.it (M.C.); antoniodimuzio1@gmail.com (A.D.M.)

**Keywords:** amyotrophic lateral sclerosis, diabetes mellitus, systematic review, glucose intolerance, hyperglycemia, metabolism, comorbidities

## Abstract

Background: Amyotrophic Lateral Sclerosis (ALS) is a degenerative disorder which affects the motor neurons. Growing evidence suggests that ALS may impact the metabolic system, including the glucose metabolism. Several studies investigated the role of Diabetes Mellitus (DM) as risk and/or prognostic factor. However, a clear correlation between DM and ALS has not been defined. In this review, we focus on the role of DM in ALS, examining the different hypotheses on how perturbations of glucose metabolism may interact with the pathophysiology and the course of ALS. Methods: We undertook an independent PubMed literature search, using the following search terms: ((ALS) OR (Amyotrophic Lateral Sclerosis) OR (Motor Neuron Disease)) AND ((Diabetes) OR (Glucose Intolerance) OR (Hyperglycemia)). Review and original articles were considered. Results: DM appears not to affect ALS severity, progression, and survival. Contrasting data suggested a protective role of DM on the occurrence of ALS in elderly and an opposite effect in younger subjects. Conclusions: The actual clinical and pathophysiological correlation between DM and ALS is unclear. Large longitudinal prospective studies are needed. Achieving large sample sizes comparable to those of common complex diseases like DM is a challenge for a rare disease like ALS. Collaborative efforts could overcome this specific issue.

## 1. Introduction

Amyotrophic Lateral Sclerosis (ALS) is a degenerative disorder of unknown cause which affects the motor neurons of the cerebral cortex, brainstem, and spinal cord [1,2,3,4].

The primary triggers for motor neuron degeneration in ALS remain elusive, however research in patients and in SOD1 mutant mice models has revealed several processes that are likely to contribute to the pathology, including inflammation and toxic glial activation [5,6], mitochondrial dysfunction [7,8], and oxidative stress [9,10].

Despite the traditional view of ALS as a pure motor neuron disorder, growing evidence suggests that ALS may impact different systems, the including cellular metabolic system [11,12,13].

Energy homeostasis requires that uptake of nutrients in cells, including glucose and lipids, is adequately controlled by the glucose–insulin axis [11].

Several studies demonstrated that two-thirds of ALS patients develop a stable hypermetabolism during the course of the disease [11,14,15,16] which has been linked to a worse prognosis in several studies [17]. However, there is no consensus in literature and, although several hypothesis were examined, the mechanisms underlying hypermetabolism are still unclear [11,14,18,19,20].

Dupuis et al. observed a protective role of hyperlipidemia in rodents with ALS and showed how a high Low Density Lipoprotein (LDL)/High Density Lipoprotein (HDL) ratio in ALS patients, which is a measure of high levels of fat storage, correlates with a better prognosis, demonstrating a favorable prognostic role of hyperlipidemia in ALS patients. These data reflect evidence that high-calorie diets improve prognosis increasing the energy and consequently reducing the energetic deficit developed in motor neurons, avoiding the development of a hypermetabolic state which particularly involves lipid metabolism. In fact, to support the increased energetic demand of denervated muscle a higher amount of free fatty acids converted through lipolysis is required [20,21].

In line with these findings, it has been observed that malnutrition negatively affects ALS patients. Moreover, a low (under-weight) pre-morbid Body Mass Index (BM) and a faster weight loss after disease onset are linked to a worse prognosis [20,22,23,24,25,26]

Contradictory findings have been reported about glucose metabolism. Some authors described the occurrence of insulin resistance during the course of ALS [27,28,29,30] and several studies investigated the role of Diabetes Mellitus (DM) as risk and/or prognostic factor [17,26,31,32,33,34,35,36,37,38,39,40,41,42,43,44,45,46,47,48,49,50].

However, it is still difficult to define a clear correlation between DM and ALS because results are conflicting and the possible underlying common pathophysiology is still elusive [13,31,44,47,48,51].

In this review, the focus is on the role of DM in ALS and different hypotheses are presented concerning how perturbations of glucose metabolism may interact with the pathophysiology and the course of the disease.

## 2. Materials and Methods

The authors undertook an independent PubMed literature search, using the following search formula: ((ALS) OR (Amyotrophic Lateral Sclerosis) OR (Motor Neuron Disease)) AND ((Diabetes) OR (Glucose Intolerance) OR (Hyperglycemia)).

**Figure 1:**
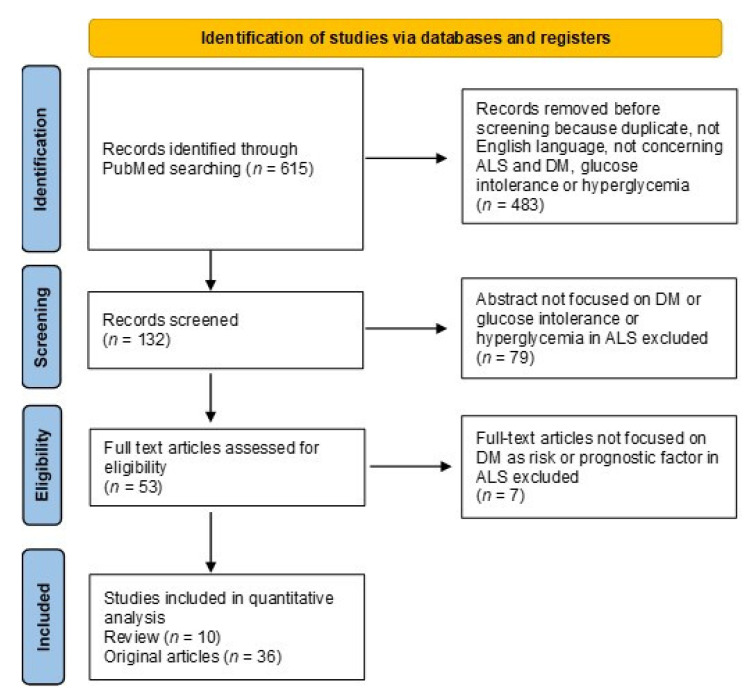
The flow-chart depicts the different phases of the literature search and analysis.

Both review and original articles were considered. The preferred reporting items for systematic reviews and meta-analyses is given in a diagram describing the research from literature (Figure 1) [52].

## 3. Results

Results reported below in narrative description are also summarized in Table 1.

### 3.1. Diabetes Mellitus as a Risk Factor for ALS

Diabetes has been studied as a risk factor for ALS, with conflicting results.

The pioneer among the studies on DM in Caucasian ALS patients is the study by Jawaid et al. [33] which found that premorbid DM2 was associated with a reduced risk of ALS and a 4 years-delayed onset compared to a group without DM2 (ALS-DM = 60.3; ALS = 56.3; *p* < 0.05) [33].

According to Jawaid et al., some authors documented a lower prevalence of DM in ALS patients compared to a control cohort matched for age and gender and this difference has been read as an expression of lower susceptibility to ALS of diabetic patients [32,36,38,39,53]. Thus, they suggested DM as a protective factor against ALS occurrence [32,36,38,39].

One potential explanation might be a selection bias which led investigators to recruit patients without comorbidities in an ALS-related clinical trial [69]. Secondly, ALS trial participants were often defined as diabetic based on self-reported medical history or retrospectively using International Classification of Disease (ICD) codes which can underestimate the prevalence of DM [41,54].

Concerning the effects of DM on age at onset of ALS, some authors reported delayed ALS onset in DM patients [39,41,49,55], while other population-based studies did not find differences of age at onset between ALS patients with or without DM [40].

Two north-European case-control studies showed that the risk of ALS was higher among subjects with a diagnosis of diabetes before the age of 40, while it was lower in those with a diabetes onset after 40 years of age [36,38].

Two recent population-based studies, the former conducted in China [42] and the latter in Taiwan [56], reported results consistent with previous studies performed in Western countries [33,55,57].

In particular, Tsai et al. performed a case-control population-based study where they found that hypometabolic disorders, especially DM, have a beneficial effect on ALS incidence [58]. After that, the same group elaborated more deeply the data about DM in ALS and they found that the onset of ALS was delayed by 4 years in DM2 patients, highlighting a protective role of DM2 on ALS onset, especially in patients older than 55 years [56].

Other studies conducted in Asian populations showed different findings. Sun et al. reported a significantly increased risk of ALS in diabetic patients compared to non-diabetics with an overall hazard ratio (HR) of 1.35. After adjustment for age, they showed an increased HR in younger diabetic men (<65 years) while for older diabetic men and women (>65 years), the risk of ALS maintained the same trend but was not significant [43,44].

In conclusion it seems that in younger patients, diabetes is a consistent risk factor for ALS. In older patients, diabetes protects against ALS in Caucasians but increases the risk in Asian populations [13].

These findings may be explained by considering the substantial differences of ALS between European and Asian populations in terms of clinical features and potential molecular mechanisms, in terms of possible biological pathways underlying ALS in different populations [37] and of the genetic basis of ALS in the two populations [37,44].

### 3.2. Diabetes Mellitus as a Prognostic Factor for ALS

Several studies investigated the association among comorbidities and ALS survival and specifically between DM and ALS. All these studies similarly reported that DM2 did not affect the course of the disease in terms of survival or in terms of ALS Functional Rating Scale Revised (ALSFRSR) and respiratory function decline [40,41,45,49,50,59].

Conversely, an assessment of the correlation with baseline glycated hemoglobin (HbA1c) and survival in a Chinese ALS cohort of patients, classified into three groups based on their HbA1c levels (<5.7%, 5.7–6.4%, and >6.4%) using the group with the lowest HbA1c level as reference, showed that increased baseline HbA1c was associated with increased risk of mortality for any cause, after adjustment for epidemiologic and clinical covariates (i.e., age, sex, site of onset, rate of disease progression, BMI, use of riluzole, gastrostomy percutaneous endoscopy, noninvasive positive pressure ventilation) [60].

Nevertheless, loss/dysregulation of energy homeostasis has been proposed to negatively impact the course of the disease with a greater LMN involvement, worse functional decline over time and reduced survival [16,17].

De la Rubia Orti et al. performed a cross-sectional study in which they investigated the role of hyperglycemia and insulin resistance index, defined as the correlation of glucose venous blood value with increased Alkaline Phosphatase (AP). A significant positive correlation was observed between insulin resistance and spinal ALS onset [61].

Regarding cognitive performance it has been reported that pre-morbid DM was associated with either similar or worse cognitive performance on neuropsychological tests at the time of ALS diagnosis [46]. Specifically, ALS-DM performed worse on tests of memory, semantic fluency, and executive function [33].

It is unclear why diabetes would have a protective effect on the motor symptoms but not on the cognitive symptoms of ALS [12].

## 4. Discussion

Despite in literature the presence of perturbations in glucose metabolism in patients with ALS has been repeatedly reported, the question about how DM and ALS interact is still open.

It is unclear if DM is a manifestation of ALS rather than a protective factor [35]. To elucidate the molecular mechanisms underlying the extra-neural pathology of ALS, some authors focus on TAR DNA-binding protein 43 kDa (TDP-43) which is found in 90–95% of sporadic ALS patients, and that in familial ALS is linked to TAR DNA-binding protein (TARDBP) or C9orf72-SMCR8 Complex Subunit (C9ORF72) genes. TDP-43 pathophysiology is linked to the loss of nuclear protein and appearance of cytoplasmatic aggregations in motor neurons [70,71].

It is known that TDP-43 can regulate whole-body metabolism and glucose transport through the obesity-related gene TBC1 domain family member 1 (Tbc1d1) [12]. Recently, it has been observed that a loss of nuclear TDP-43 underlies the impaired early-phase secretion of insulin which is observed in early-stage ALS patients [12,72,73,74]. Araki et al. proposed a possible role for nuclear TDP-43 depletion in pancreatic β cells in the decreased insulin secretion of patients with sporadic ALS [73].

Moreover, it has been observed that SOD1 mice exhibit signs of metabolic dysfunction even at the pre-symptomatic stage of ALS, with dysregulation of lipid and carbohydrate metabolism [19,20].

An exon-array analysis showed that C9ORF72 repeat expansions lead to a differentially regulated splicing of several genes involved in cholesterol biosynthesis and glucose metabolism [12,75,76,77].

Furthermore, some authors speculated that the protective role of DM on ALS onset might be due to the effect of anti-diabetic drugs [62]. More specifically, two different studies investigated the role of pioglitazone and metformin in patients with ALS. Dupuis et al. reported a phase II, multicentric, placebo-controlled trial of pioglitazone in ALS patients under riluzole treatment regimen. Pioglitazone is used in clinical practice as an anti-diabetic agent; however, it also plays a role as an anti-oxidant and anti-inflammatory agent, which made it a candidate for ALS treatment since oxidative stress and inflammation are implicated in ALS pathophysiology [78]. Pre-clinical studies in SOD1 transgenic mice showed positive results [79,80,81].

Notwithstanding the promising pre-clinical data, Dupuis et al. had to stop that trial for futility, in absence of effects on survival and secondary outcomes as ALSFRS-R score and decline of respiratory functions [63].

Similarly, Kaneb et al. performed a pre-clinical study with metformin in SOD1 transgenic mice on the basis of its anti-inflammatory [82,83,84] and anti-oxidant [85,86] properties [87]. They observed a lack of efficacy on survival in male mice and a negative effect on onset and progression in female mice. Therefore they suggest that metformin is a poor candidate for clinical trials in ALS patients [64].

Concerning pioglitazone, it has been suggested that the lack of efficacy could be due to the interaction between beneficial molecular mechanism of the drug and this opposite detrimental effect on the whole-body metabolism, i.e., weight loss which is associated with a faster disease progression [57,65]. Moreover, some authors speculated that the unexpected failure of anti-diabetic drugs in ALS might be due to the nullification of the supposed beneficial effects of DM-associated hypermetabolism in ALS patients [65].

In fact, the third mechanism proposed is that high levels of glucose in ALS patients could reduce the damage caused to motor neurons by hypermetabolism.

Hypermetabolism is defined as a significant increase in measured resting energy expenditure (REE) relative to predicted REE, based on the Harris–Benedict equation [14,15,20,88] or measurements including fat-free mass [16].

Several studies suggested that in an attempt to avoid an increase in oxidative stress in the pre-symptomatic stage of the disease [11,15,39], mitochondria become uncoupled, ensuing in hypermetabolism [89,90]. Hence, higher resting metabolism could be activated to maintain energetic status [13].

This hypermetabolism may appear paradoxical because Free Fat Mass (FFM), the main determinant of REE, decreases in ALS [14,15,88]. Although many hypotheses have been proposed, such as increased respiratory muscular work [88], hypothalamic involvement [16], hyperthyroidism, spasticity or fasciculation intensity [18], hypermetabolism remains largely unexplained.

As above mentioned, several studies reported that hypermetabolic patients with ALS have a trend towards shorter survival [16,91]. Thus, hypermetabolism has been indicated to be a negative prognostic factor for ALS.

In this context, some authors speculated that DM positive effects should be the results of the activation of alternative pathways, such as ketogenesis, which are metabolically more efficient than glucose or fats in mitochondrial ATP generation [92,93,94].

Moreover, Jawaid et al. suggested that because the formation of stress granules might be induced by glucose deprivation, factors that lead to glucose starvation might enhance formation of TDP-43/FUS aggregation, and conversely DM2 should provide protection by supplying more glucose or alternate sources of energy preventing formation of stress granules [12]. Further, it has been proposed that DM could exert its protective role through hyperglycemia, which has been described to promote glutamate uptake [36,95].

Indeed, it has been demonstrated that a high-caloric diet, with either high carbohydrate or high fat contents, improves prognosis [20,24,66,96,97,98] increasing energy availability and consequently reducing the energy deficit developed in motor neurons [20].

Nevertheless, contrasting data, resulting from the analysis of glucose pathway, underline how abnormalities in the course of glucose metabolism pathway can lead to decreased generation of adenosine triphosphate (ATP) and a subsequent activation of compensatory alternative energy generating pathways which may result in increased oxidative stress [51,99,100,101].

Furthermore, some authors suggested that hypermetabolism may not be a direct factor influencing prognosis, but rather, a consequence of the rearrangement of whole-body metabolism and the activation of less efficient energy pathways due to enhanced request or absence of metabolic substrate during the course of the disease, often present due to malnutrition as a result of dysphagia, inability of feeding oneself, fear of choking, and aspiration pneumonia [14,22,91,102].

Fourth, it is possible that the deep differences in natural disease histories of DM and ALS may confound the reported low prevalence of DM in ALS. Indeed, it is notable that hyperlipidemia, which has been shown to have a protective role in ALS [21], is frequent in DM patients, while a slim body and athletic life-style, suggested as risk factors for ALS, are uncommon in DM patients [4,103,104]. Hence, DM patients could have a less malignant course of ALS because of either the presence of a protective factor (hyperlipidemia) or absence of certain risk factors (low BMI and active lifestyle) [33].

Finally, Apolipoprotein E (APoE) has been proposed to be involved in the pathophysiology of ALS and to influence the course of ALS. ApoE is the key regulator of plasma lipids, mediating altered functions in lipoprotein metabolism. ApoE genotype has been observed to be implicated as a risk factor in several neurological disorders such as Alzheimer’s Disease, Creutzfeldt-Jacob disease, Wilson’s disease, Multiple Sclerosis, cerebral amyloid angiopathy, Parkinson’s Disease and Lewy Body Dementia [105,106,107,108,109,110,111,112,113]. It has been speculated that its impact in neurological diseases is due to its role in lipid transport, neuronal repair, calcium homeostasis, and antioxidant activity [66,88,89,90,96].

Some studies observed a higher age of onset of ALS in patients carrying the ApoE2 allele [114,115], while a lower age of onset was detected in subjects with Apoe4 allele [116,117]. However, no correlation with the clinical course of ALS was detected in association with the ApoE genotype [67,118]. ApoE plasma levels have been correlated with a worse rate of physical deterioration and shorter survival time [118].

Both ApoE2 and ApoE4 have been found to be over-expressed in diabetic and hyperlipidemic populations in North America [119]. Consequently, some authors suggested that the supposed beneficial effects of DM on ALS are due to the ApoE genotype which may independently influence the risk of developing DM and the onset and progression of ALS [33].

## 5. Conclusions

According to our systematic review, DM appears not to affect disease severity, progression, and survival in ALS patients. On the other hand, contrasting data suggested a protective role of DM on the occurrence of ALS in elderly subjects and an inverse correlation in younger. But, there is still no consensus and the real clinical and pathophysiological correlation between DM and ALS is unclear. We describe how the multiple attempts to shed light on the relationship between DM and ALS may be hindered by several shortcomings.

The major limit of the above-mentioned studies is the difficulty in designing a study considering the dynamic course of DM, including measures of glycaemia control, and taking into account the wide range of co-variables associated with DM, as short and long term DM complications, in patients with ALS, which should partially justify the heterogeneity of currently available data.

On this basis we believe that it could be useful, in further studies, to assess an accurate distinction between DM1 and DM2 [68]. Indeed, many authors identified diagnosis of DM using International Classification of Disease (ICD) codes extrapolated from registers which enabled them to classify DM in type 1 and type 2 sometimes only based on insulin dependence [33,36,38,39]. This inaccurate distinction is relevant because available data suggest a different interaction between ALS and DM1 and DM2, explained by distinct pathophysiology of DM1 which recognize an autoimmune genesis, notably associated to an increase risk of ALS [120].

In this regard, Kioumourtzoglou et al. reanalysed previously collected data and re-classified part of their DM2 cohort as DM1, and found an overall protective association between DM2 and ALS diagnosis, while DM1 would represent a risk factor for ALS [36].

Moreover, Jawaid suggested that not only the pathophysiology should be considered but also the differences in body phenotype between DM1 and DM2. In particular, DM1 is more often associated with weight loss, which is associated with a faster ALS progression [33].

Despite discrepancies and limits of the available studies, considering the growing evidence of insulin-resistance involvement also in other neurodegenerative diseases such as Alzheimer’s Disease and Parkinson’s Disease [121,122,123,124], we suggest that further efforts in determining the role of glucidic metabolism in ALS would be crucial. Therefore, to obtain more reliable results, large longitudinal prospective studies are needed.

Notably, achieving large sample sizes comparable to those of common complex diseases like DM is a challenge for a rare disease like ALS. Collaborative efforts would hopefully overcome this specific challenge.

## Figures and Tables

**Table 1:** Summary of the correlation between main issue and ALS course and prognosis.

First Author, Year References	Type of Study	Nation	Main Issue	Focus on	Presence of Association	Sample Size	Effect Size
Dupuis, 2011 [11]	Review	-	Metabolism (glucose intolerance, DM)	-	-	-	-
Jawaid, 2018 [12]	Review	-	Metabolic Perturbations (DM)	-	-	-	-
Brito, 2019 [13]	Review	-	Metabolism (DM)	-	-	-	-
Vucic, 2018 [17]	Editorial	-	Hypermetabolism (glucose metabolism)	-	-	-	-
Pape, 2020 [23]	Review	-	Nutritional Status	-	-	-	-
Jawaid, 2010 [25]	Cohort	USA	DM, BMI	Age Of Onset (years)	yes	274 ALS	ALS-DM 60.8 ± 10.0 vs. ALS 52.0 ± 13.5 (*p* = 0.01)
Pradat, 2010 [27]	Case-control	France	Glucose Intolerance	Prognosis (months)	no	21 ALS vs. 21 controls	17.4 ± 11.4 vs. 20.4 ± 10.7 (*p* = 0.62)
Harno, 1984 [28]	Case-control	Finland	Glucose Intolerance, DM, OGTT	-	-	21 ALS vs. 10 controls	-
Reyes, 1983 [29]	Case-control	USA	Glucose Intolerance	-	-	10 ALS vs. 19 healthy controls vs. 4 neuromuscular controls	-
Shimizu, 2011 [30]	Short report	Japan	Glucose Intolerance	-	-	-	-
Lekoubou, 2014 [31]	Review	-	DM	-	-	-	-
D’Ovidio, 2018 [32]	Cohort	Italy	DM	Risk	yes	727,977 general population (24 ALS-DM vs. 373 ALS)	HR 0.30 (95% CI 0.19–0.45)
Jawaid, 2010 [33]	Cohort	USA	DM	Age Of Onset (years)	yes	ALS-DM 175 vs. ALS 2196	60.3 vs. 56.3 (*p* < 0.05)
				NTP	yes		Animal fluency (*p* = 0.03), Logical memory (*p* = 0.01), Trails A-B (*p* = 0.05)
Jawaid, 2015 [34]	Letter to the editor	-	DM	-	-	-	-
Jawaid, 2015 [35]	Editorial	-	DM	-	-	-	-
Kioumourtzoglou, 2015 [36]	Nested case-control	Denmark	DM	Risk	yes	3650 ALS vs. 365,000 controls	OR 0.61 (95%, CI 0.46–0.80)
Logroscino, 2015 [37]	Editorial	-	DM	-	-	-	-
Mariosa, 2015 [38]	Case-control	Sweden	DM	Risk	yes	224 ALS vs. 1437 controls	OR 0.79 (95% CI 0.68–0.91)
Mariosa, 2020 [39]	Case-control	Sweden	Antidiabetic Drugs (DM)	Risk	yes	2475 ALS vs. 12,375 controls	OR 0.76 (95% CI 0.65–0.90)
Moglia, 2017 [40]	Cohort	Italy	Comorbidities (DM)	Age Of Onset (years [SD])	no	52 ALS-DM vs. 598 ALS	66.8 (8,2) vs. 66.4 (10,8), *p* = 0.76
				Survival (years)	no		2.77 (95% CI 1.6–4.2) vs. 2.5 (95% CI 1.5–4.3), *p* = 0.84
Paganoni, 2015 [41]	Cohort	USA	DM	Prognosis	no	71 ALS-DM vs. 1251 ALS	not provided
Zhang, 2019 [42]	Cohort	China	DM	Age Of Onset (years [SD])	yes	100 ALS-DM vs. 1231 ALS	57.0 (9.6) vs. 52.6 (10.3), *p* = 0.000
				Prognosis	no		HR 0,84 (95% CI 0.68–1.30, *p* = 0.617)
Sun, 2015 [43]	Cohort	Taiwan	DM	Risk	yes	615,492 DM vs. 614,835 no-DM (255 ALS-DM vs. 201 ALS)	HR 1.35 (95% CI 1.10–1.67)
Zeng, 2019 [44]	Case-control	Europe-Asia	DM	Risk	yes	Europe: 660,000 ALS-DM vs. 81,000 ALS Asia: 191,000 ALS-DM vs. 4100 ALS	Europe OR 0.93 (95% CI 0.88–0.99 *p* = 0.023) Asia: OR 1,28 (95% CI 0.99–1.62 *p* = 0.058)
Mandrioli, 2018 [45]	Cohort	Italy	Comorbidities (DM)	Prognosis	no	230 ALS-DM vs. 2124 ALS	HR 1.11 (95% CI 0.93–1.33, *p* = 0.259)
Vasta, 2021 [47]	Review	-	DM	-	-	-	-
Wannarong, 2020 [48]	Review and Meta-analysis	-	DM	-	-		-
Schumacher, 2020 [49]	Cohort	Germany	DM	Age Of Onset (years)	yes	59 ALS-DM vs. 442 ALS	69.1 ± 8.6 vs. 64.7 ± 11.1 (95% CI 63.7–65.8)
				Prognosis	no		HR 1.05 (95% CI 0.74–1.49, *p* = 0.8)
Bellavia, 2020 [50]	Nested case-control	USA	Comorbidities (DM), Environment	Risk	no	1086 ALS	not provided
Tefera, 2021 [51]	Review	-	Glucose Metabolism	-	-	-	-
Mariosa, 2017 [53]	Longitudinal cohort	Sweden	Hyperglycemia	Risk	yes	254 ALS vs. 635,878 controls	HR 0.62 (95% CI 0.42–0.93)
Mitchell, 2015 [54]	Case-control	USA	Comorbidities (DM)	Risk (Prevalence)	yes	1288 ALS vs. 7561 controls	ALS-DM 7.38%, DM 14.47%; OR 0.47
Hollinger, 2016 [55]	Case-control	USA	Comorbidities (DM)	Age Of Onset (years)	no	129 ALS-DM vs. 1310 ALS	50.9 ± 14.4 vs. 56.0 ± 13.4 (*p* = 0.1439)
				Prognosis	no		OR 1.18 (0.699–1.992)
Tsai, 2019 [56]	Population-based cohort	Taiwan	DM	Risk	no	2,135,427 DM vs. 2,135,427 controls (195 ALS-DM vs. 210 ALS)	HR 0.87 (95% CI 0.7–1.07)
				Risk (DM onset < 55 yo)	no		HR 1.24 (95% CI 0.88–1.75)
				Risk (DM onset > 55 yo)	yes		HR 0.72 (95% CI 0.55–0.95, *p* = 0.019)
Jawaid, 2018 [57]	Editorial	-	DM	-	-	-	-
Tsai, 2018 [58]	Case-control	Taiwan	Comorbidities (DM)	Risk	yes	705 ALS vs. 14,100 controls	OR 0.7 (*p* < 0.05)
Korner, 2013 [59]	Cohort	Germany	Comorbidities (DM)	Prognosis	no	37 ALS-DM vs. 477 ALS	HR 1.18 (95% CI 0.81, 1.70, *p* = 0.39)
Wei, 2017 [60]	Cohort	China	Hyperglycemia	Prognosis	yes	152 HbA1c 5.7–6.4%	HR 1.4 (95% CI 1.02–1.99)
				Prognosis	yes	35 ALS HbA1c > 6.5%	HR 2.06 (95% CI 1.07–3.96)
De la Rubia Orti, 2021 [61]	Case-control	Spain	Insulin Resistance	Phenotype	yes	20 spinal onset	Rho 0.695, (*p* = 0.011)
					no	11 bulbar onset	Rho 0.001 (*p* = 0.987)
					no	29 controls	Rho 0.187 (*p* = 0.252)
Pfeiffer, 2019 [62]	Case-control	USA	Antidiabetic Drugs (DM)	Risk	yes (HRG)	10,450 ALS vs. 104,500 controls	OR 0.73 ± 0.06 (*p* < 0.05)
Dupuis, 2012 [63]	RCT	Germany	Antidiabetic Drugs (DM)	Survival (Pioglitazone vs. Placebo)	no	219 ALS (one:one allocation ratio)	HR 1.21 (95% CI 0.71–2.07, *p* = 0.48)
Kaneb, 2011 [64]	Animal study	UK	Antidiabetic Drugs (DM)	Survival (days) (control vs. Metformin 0.5 mg/mL vs. 2 mg/mL vs. 5 mg/mL)	no	14 SOD1 male mice for each group	123 vs. 128 vs. 126 vs. 126, *p* = 0.8575
					no	15 SOD1 female mice for each group	140 vs. 136 vs. 132 vs. 132, *p* = 0.6216
Jawaid, 2014 [65]	Review	-	Antidiabetic Drugs (DM)	-	-	-	-
Ferri, 2017 [66]	Review	-	Hypermetabolism (glucose metabolism)	-	-	-	-
Jawaid, 2011 [67]	Cohort	USA	ApoE Genotypes	Age Of Onset (years)	no	852 ALS (E2 = 93 vs. E3 = 537 vs. E4 = 212)	57.5 ± 13.5 vs. 57.3 ± 13.7 vs. 57.7 ± 13.2, (*p* = 0.97)
				Survival (years)	no		3.79 ± 3.7 vs. 3.16 ± 2.38 vs. 3.05 ± 1.75, (*p* = 0.85)
Kawada, 2015 [68]	Letter to the editor	-	DM	-	-	-	-

DM: Diabetes Mellitus; ALS: Amyotrophic Lateral Sclerosis; ALS-DM: patients affected by both ALS and Diabetes Mellitus; OGTT: Oral Glucose Tolerance Test; NTP: neuropsychological test performance; Trails A&B: trails making test parts A&B; HRG: human recombinant Glucagon; ApoE: Apolipoprotein E; OR: Odds Ratio; HR: Hazard Ratio; 95% CI: 95% Confidence Intervals; SD: Standard Deviation; RCT: Randomized Clinical Trail; HbA1c: Glycated Hemoglobin.

## Data Availability

Not applicable.
